# Quantifying substantial carcinogenesis of genetic and environmental factors from measurement error in the number of stem cell divisions

**DOI:** 10.1186/s12885-022-10219-w

**Published:** 2022-11-19

**Authors:** Xinhui Liu, Jifeng Yang, Hongkai Li, Qing Wang, Yuanyuan Yu, Xiaoru Sun, Shucheng Si, Lei Hou, Lu Liu, Fan Yang, Ran Yan, Yifan Yu, Zhentao Fu, Zilong Lu, Dejin Li, Hao Xue, Xiaolei Guo, Fuzhong Xue, Xiaokang Ji

**Affiliations:** 1 Department of Biostatistics, School of Public Health, Cheeloo College of Medicine, Shandong University, PO Box 100, 44 Wenhuaxi RoadShandong Province, Shandong 250012 Jinan, China; 2 Institute for Medical Dataology, Cheeloo College of Medicine, Shandong University, Jinan, Shandong 250012 China; 3 Shandong Health Care Industry Association, Jinan, Shandong 250002 China; 4 Shandong Center for Disease Control and Prevention, Jinan, Shandong 250014 China; 5 Shandong Provincial Big Data Center, Jinan, Shandong 250011 China; 6 Department of Neurosurgery, Qilu Hospital, Cheeloo College of Medicine, Shandong University, Jinan, Shandong 250012 China

**Keywords:** Cancer prevention, Environment, Stem cell division, Measurement error, Epidemiology

## Abstract

**Background:**

The relative contributions of genetic and environmental factors versus unavoidable stochastic risk factors to the variation in cancer risk among tissues have become a widely-discussed topic. Some claim that the stochastic effects of DNA replication are mainly responsible, others believe that cancer risk is heavily affected by environmental and hereditary factors. Some of these studies made evidence from the correlation analysis between the lifetime number of stem cell divisions within each tissue and tissue-specific lifetime cancer risk. However, they did not consider the measurement error in the estimated number of stem cell divisions, which is caused by the exposure to different levels of genetic and environmental factors. This will obscure the authentic contribution of environmental or inherited factors.

**Methods:**

In this study, we proposed two distinct modeling strategies, which integrate the measurement error model with the prevailing model of carcinogenesis to quantitatively evaluate the contribution of hereditary and environmental factors to cancer development. Then, we applied the proposed strategies to cancer data from 423 registries in 68 different countries (global-wide), 125 registries across China (national-wide of China), and 139 counties in Shandong province (Shandong provincial, China), respectively.

**Results:**

The results suggest that the contribution of genetic and environmental factors is at least 92% to the variation in cancer risk among 17 tissues. Moreover, mutations occurring in progenitor cells and differentiated cells are less likely to be accumulated enough for cancer to occur, and the carcinogenesis is more likely to originate from stem cells. Except for medulloblastoma, the contribution of genetic and environmental factors to the risk of other 16 organ-specific cancers are all more than 60%.

**Conclusions:**

This work provides additional evidence that genetic and environmental factors play leading roles in cancer development. Therefore, the identification of modifiable environmental and hereditary risk factors for each cancer is highly recommended, and primary prevention in early life-course should be the major focus of cancer prevention.

**Supplementary Information:**

The online version contains supplementary material available at 10.1186/s12885-022-10219-w.

## Background

Cancer registries show striking variation in cancer incidence across different tissues. For instance, based on the Surveillance, Epidemiology and End-Results (SEER) Cancer Registry from 2015 to 2017 (https://seer.cancer.gov/statfacts/more.html), prostate cancer is around 125 times more frequently diagnosed than bone and joint cancer. Three factors: inherited genetic variation, external environmental factors, and the stochastic effects associated with the lifetime number of stem cell divisions within each tissue, have been shown to explain the variation in organ-specific cancer risk, however, the relative contributions of hereditary and environmental factors versus unavoidable stochastic risk factors have become a widely-discussed topic, which is important not only for understanding this disease but also for designing strategies to reduce the cancer mortality.

In 2015, *Tomasetti and Vogelstein *[1] reported a strong positive correlation between the total number of stem cell divisions during the average lifetime of a human within each tissue and the tissue-specific lifetime cumulate risk for cancer, and drew the conclusion that “bad luck”, i.e., the stochastic mutations arising during DNA replication in normal stem cells, has a major role in cancer development, only a third of the variation in cancer risk among tissues attributes to environmental factors or inherited predispositions. Besides, this study indicated that for cancers that stochastic factors play a relatively more important role in their risk, the main strategy to reduce cancer deaths should be secondary prevention. This original finding had sparked controversy and caused reaction through research papers, some of which came to opposite conclusions [2–4]. In 2017, *Tomasetti, Li, and Vogelstein* [5] expanded their data to 423 cancer registries in 69 countries throughout the world, and found universal strong correlations between the number of normal stem cell divisions and cancer incidences in all countries, regardless of large differences in exposures to environmental factors and associated cancer incidences, this further approved their previous conclusions.

However, as pointed out by *Perduca *et al. [6], the estimation of the number of lifetime stem cell divisions in studies of *Tomasetti, Li and Vogelstein* [1, 5] did not account for specific genetic and environmental factors that possibly influence both the total number of stem cells in an organ (parameter *s*) and the lifetime number of divisions of each stem cell (parameter *d*). In other words, the implicit assumption underpinning the universally high correlations between the number of normal stem cell divisions and cancer risk in all 69 countries is that the number of normal stem cell divisions is not affected by genetic and environmental factors, i.e., the number of normal stem cell divisions in a given tissue basically do not change facing large differences in exposures to environmental factors and associated cancer incidences across the world.

Given that such an assumption is unrealistic, *Perduca *et al*.* [6] indicated that it is reasonable to assume an indirect effect of environmental and genetic factors on cancer risk possibly mediated by the number of stem cell divisions. Those modelling approaches proposed by *Wu *et al*.* [2] were based on a similar assumption. *Wu *et al*.* [2] assumed that cancers with similar number of stem cell divisions should share the same base of intrinsic cancer risk, and the differences between the incidence of these cancers should be attributed to additional (probably extrinsic) risk factors [2]. Therefore, they obtained an “intrinsic” risk line by regressing the smallest cancer risks on the corresponding number of stem cell divisions (Fig. 3a in *Wu *et al. [2], red line), the excess risks of other cancers (above this line) implied large proportions of risks that may not be attributed to intrinsic stochastic factors (mostly larger than 90%), this indicated that intrinsic stochastic risk factors contribute only modestly to cancer development [2]. However, their study also sparked debates through many research papers [4, 7].

In this study, we investigated the contribution of hereditary and environmental factors to the variation in organ-specific cancer risk from a new perspective. We also assumed that the environmental and genetic factors have an indirect effect on cancer risks mediated by the number of stem cell divisions. The true value of the total number of stem cell divisions in the lifetime of a tissue varies with the exposure to different levels of genetic and environmental factors (roughly represented by different registries throughout the world) is almost impossible to be obtained. We can only roughly calculate the number of stem cell divisions using parameter *s* and *d* estimated based on results of cell culture from mouse or human tissues in the laboratory environment that is cleaner than any registry throughout the world. Theoretically speaking, for any registry, the calculated number of stem cell divisions should be different from its true value, this can be regarded as a measurement error [8] problem.

From this point of view, the universally strong correlations between the number of normal stem cell divisions and cancer risk in all countries [5] implies the ignorance of the measurement errors in the estimated number of stem cell divisions. These errors are caused by the exposure to different levels of genetic and environmental factors that do not exist in the laboratory environment, the estimated correlations without considering measurement errors may be seriously biased. Although several studies [1, 2, 5] also conducted sensitivity analyses with the consideration of the uncertainty for the estimation of the number of stem cell division by assuming a 100-fold variation (plus and minus), it is less likely that the measurement error in the estimated number of stem cell divisions subjects to a classical additive measurement error model (the estimated value is distributed with independent random error around the true value) [8]. Since these measurement errors are caused by additional (mostly unknown) hereditary and environmental factors that do not exist in the laboratory environment but affect mutagenesis rates, the ignorance of such measurement error is certain to obscure the authentic contribution of hereditary and environmental factors to the variation in organ-specific cancer risk. In addition, we hold the opinion that the contribution of random mutations arising during DNA replication in normal stem cells to the variation in cancer risk cannot be measured by Pearson correlation coefficient (*r*) and its square (*r*^*2*^) on a log–log scale, because the log transformation actually narrows the variances of variables and hides their original variation.

In this study, we proposed two distinct modeling strategies, which integrate the measurement error model with the prevailing model of carcinogenesis, to quantitatively evaluate the contribution of hereditary and environmental factors to cancer development.

## Methods

### Variation in cancer risk among tissues explained by genetic and environmental factors

**Fig. 1:**
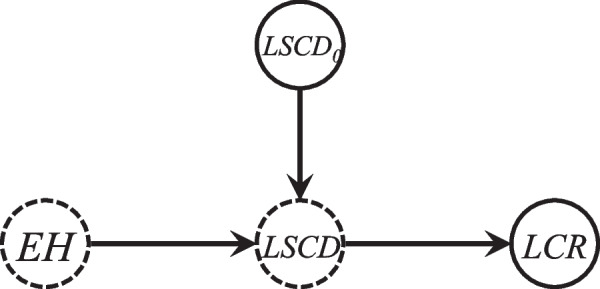
Diagram of relationships between *EH*, *LSCD*_*0*_, *LSCD*, and *LCR*. The dotted node represents the unobserved variable, and the solid node represents observed variable. *EH*: a single variable denoting all the genetic and environmental factors that do not exist in the laboratory environment; *LSCD*: the true total number of divisions of all stem cells within this tissue per lifetime (from birth to age 74); *LSCD*_*0*_: the error-prone value of *LSCD* calculated using parameters estimated based on results of cell culture from mouse or human tissues in the laboratory environment; *LCR*: the observed lifetime cancer risk

In this section, we proposed a modeling strategy to explore the contribution of genetic and environmental factors to the variation in cancer risk among tissues. We assumed that the main biological cause of cancer is the accumulation of cell divisions in stem cells, which drives the accumulation of the DNA alterations required for carcinogenesis as well as the formation and growth of the abnormal cell populations required for cancer to occur, these cell divisions are caused by the sum of a variety of physiological, pathological, and environmental factors [9]. For any tissue we considered in this study, let *LSCD* denote the true total number of divisions of all stem cells within this tissue per lifetime (from birth to age 74), *LCR* denote the lifetime cancer risk, and *EH* be a single variable denoting all the genetic and environmental factors that do not exist in the laboratory environment. *EH* may include all lifespan external environmental exposure factors and a few hereditary factors which promote the accumulation of stem cell divisions by acting on stem cells or the stem cell environment, as well as the interaction of these factors (e.g., gene–gene or gene-environment interactions) [9]. Since the true *LSCD* almost impossible to be obtained, let *LSCD*_*0*_ denote the error-prone value of *LSCD* calculated using parameters estimated based on results of cell culture from mouse or human tissues in the laboratory environment, i.e., *LSCD*_*0*_ can reflect the basic number of stem cell divisions with unavoidable stochastic risk factors under the laboratory environment, which environment is better than any registry (county) throughout the world. Therefore, in each registry (county), the measurement error in *LSCD*_*0*_ (i.e., the difference between *LSCD* and *LSCD*_*0*_) all attributes to its corresponding *EH*. We note that our modeling strategy may underestimate the contribution of all the environmental and genetic factors, because the defined *EH* does not involve factors that also exist in the laboratory environment, therefore this part of the effect cannot be estimated. Then the diagram illustrating the relationships between these variables was given in Fig. 1.

The *LCR* of 17 cancer types, including head and neck squamous cell carcinoma, esophageal squamous cell carcinoma, colorectal adenocarcinoma, hepatocellular carcinoma, pancreatic ductal/endocrine cancer, lung adenocarcinoma, osteosarcoma, melanoma, ovarian germ cell carcinoma, testicular germ cell carcinoma, medulloblastoma, thyroid follicular/papillary carcinoma, thyroid medullary carcinoma, chronic lymphocytic leukemia, acute myeloid leukemia, prostate cancer, breast cancer were calculated in 423 registries in 68 different countries (global-wide), 125 registries across China (national-wide of China), and 139 counties in Shandong province (Shandong provincial, China), respectively, using data from the International Agency for Research on Cancer (IARC), Chinese Cancer Registry Annual Report, cancer registration database from Shandong Center for Disease Control and Prevention, and population information database from Shandong Provincial Big Data Center. The *LSCD*_*0*_ of these tissues were calculated based on parameters from the supplementary materials in *Tomasetti and Vogelstein* [1, 5]. The details were provided in the Supplementary Method 1, Additional File 1.

**Fig. 2:**
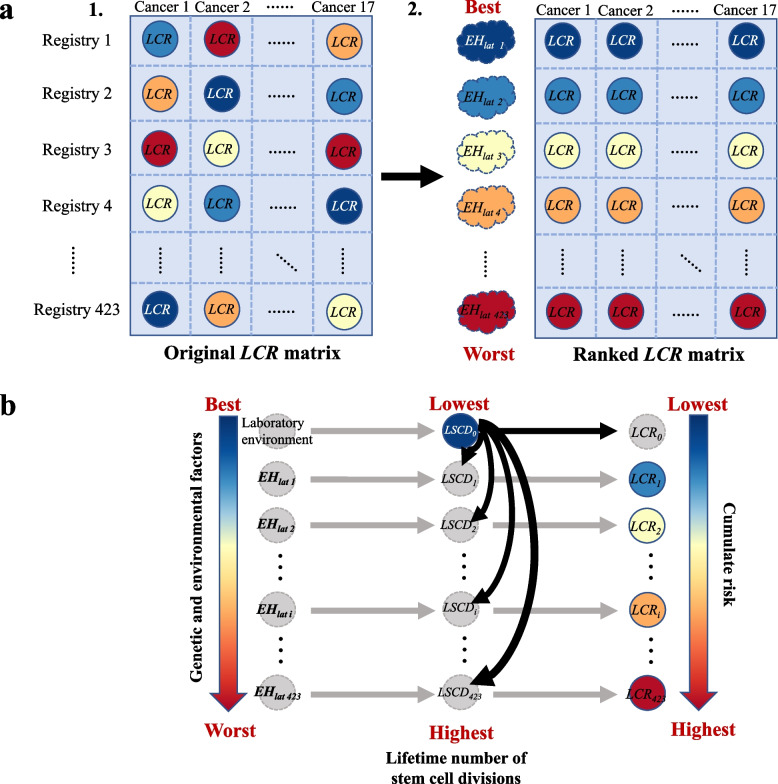
Strategy to explore the contribution of genetic and environmental factors on variation in cancer risks. **a** The construction of the ranked lifetime cancer risk (*LCR*) matrix. Part 1: The 423 × 17 original *LCR* matrix using global-wide data for 17 cancer types in 423 registries in 68 different countries. For the *j*^*th*^ column in the matrix, the color from dark blue to dark red represents the *LCR* of cancer *j* ranges from the lowest to the highest in 423 registries, and it can also indirectly denote the *EH* in 423 registries of cancer *j* ranged from the best to the worst. Part 2: The ranked *LCR* matrix, constructed by sorting the *LCR* in each column (each cancer) in the original *LCR* matrix from the lowest to the highest. We denote the level of *EH* (row) in the ranked *LCR* matrix as *EH*_*lat*_, i.e., the first row of the ranked *LCR* matrix is regarded as the optimal *EH*_*lat*_ of all 17 cancers in 423 registries, followed by the second optimal *EH*_*lat*_, until to the worst *EH*_*lat*_. **b** For the *i*^*th*^* EH*_*lat*_ (row) in the ranked *LCR* matrix, the *LCR*_*i*_ attributes to the *i*^*th*^* LSCD*_*i*_, which is caused by the *i*^*th*^* EH*_*lat i*_. However, the true number of stem cell divisions under each *EH*_*lat*_ ($LSCD_{1} , \cdots ,LSCD_{{{423}}}$) cannot be observed, we can only obtain *LSCD*_*0*_ under the laboratory environment. Dotted grey nodes represent the unmeasured variables

We taking the global-wide data of *LCR* for 17 cancer types in 423 registries in 68 different countries as an example to illustrate the rationale of the proposed modelling strategy. Those *LCR* data can be organized into an $423\times 17$ original *LCR* matrix (shown in Fig. 2a.1), the element ${LCR}_{ij}, i \epsilon \left\{1,\cdots ,423\right\}, j \epsilon \left\{1,\cdots ,17\right\}$ denotes the *LCR* for cancer *j* in the *i*^*th*^ registry. In practice, genetic and environmental factors and their exposure levels (*EH*) are different across different cancer types in each registry and different registries within each organ-specific cancer. Although *EH* is a latent variable that cannot be observed, according to the diagram of relationships between *EH*, *LSCD*_*0*_, *LSCD*, and *LCR* in Fig. 1, the worsening of the latent *EH* will primarily promote the division number of stem cells (*LSCD*) and finally increase the cancer risk (*LCR*), i.e., the higher the *LCR*, the worse the *EH*. Therefore, this original *LCR* matrix corresponds to a latent original *EH* matrix with the same dimension (for the *j*^*th*^ column in the matrix in Fig. 2a.1, the color from dark blue to dark red represents the *LCR* of cancer *j* ranges from the lowest to the highest in 423 registries, and it can also indirectly denote the *EH* in 423 registries of cancer *j* ranged from the best to the worst. Here, the best (worst) *EH* is defined as *EH* with the minimum (maximum) risk factor or exposure level that lead to the lowest (highest) cancer risk). Then, we constructed a ranked *LCR* matrix by sorting the *LCR* in each column (each cancer) in the original *LCR* matrix from the lowest to the highest (Fig. 2a.2), which can indirectly denote the latent *EH* of each cancer ranked from the best to the worst. From the point of view of the whole ranked matrix, the first row represents the respective optimal *EH* of each cancer in 423 registries, followed by the respective second optimal *EH*, lasting to the respective worst *EH*. We denote the level of *EH* (row) in the ranked *LCR* matrix as *EH*_*lat*_, i.e., the first row of the ranked *LCR* matrix is regarded as the optimal *EH*_*lat*_ of all 17 cancers in 423 registries, followed by the second optimal *EH*_*lat*_, until to the worst *EH*_*lat*_.

For the *i*^*th*^* EH*_*lat*_ (row) in the ranked *LCR* matrix, the *LCR*_*i*_ attributes to the *i*^*th*^* LSCD*_*i*_, which is caused by the *i*^*th*^* EH*_*lat*_ (Fig. 2b). However, the true number of stem cell divisions under each *EH*_*lat*_ ($LSCD_{1} , \cdots ,LSCD_{{{423}}}$) cannot be observed, we can only obtain *LSCD*_*0*_ under the laboratory environment. Then, for *i*^*th*^* EH*_*lat*_ (row) in the ranked *LCR* matrix, we fitted a curve using *LCR*_*i*_ and *LSCD*_*0*_ of 17 cancer types in their original scale.

$$g(LCR_{i} ) = \varphi_{0i} + \varphi_{1i} LSCD_{{0}} + \varepsilon_{1i} ,$$

with link function $g(x): = x^{1/5}$ aiming at normalizing variable *LCR*_*i*_, parameters in this model were estimated using nonlinear least squares. We calculated the coefficient of determination ($R_{i}^{2}$) from the curve on the original scale of *LCR*_*i*_ by the following equation.

$$R_i^2=1-\frac{\sum{(LCR_i-L\widehat CR_i)^2}}{\sum{(LCR_i-L\overline CR_i)^2}},$$

where $L\overline{C}R_{i}$ is the mean of observed data *LCR*_*i*_, $L\hat{C}R_{i}$ is the fitted value. The $R_{i}^{2}$ suggests the proportion of the differences in cancer risk among different tissues in the *i*^*th*^* EH*_*lat*_ that can be explained by *LSCD*_*0*_. Therefore, $1 - R_{i}^{2}$ can approximately and indirectly measure the contribution of the measurement error (caused by *EH*_*lat i*_) in *LSCD*_*0*_, it can also be interpreted as the contribution of genetic and environmental factors in the *i*^*th*^* EH*_*lat*_. We further conducted simulation studies to validate this modeling strategy, see Supplementary Method 2, Additional File 1 for the details.

Based on this modeling strategy, we constructed three ranked *LCR* matrixes using global-wide (covering 423 registries in 68 different countries), national-wide of China (125 registries across China), and its Shandong provincial (139 counties in Shandong province) data of *LCR* for 17 cancer types, we fitted the model by 423, 125, and 139 times respectively, and calculated $1 - R_{{}}^{2}$ for each model.

Our proposed modelling strategy is based on the assumption that cancers originate in stem cells, which may not be wholly accepted. Therefore, we also conducted a sensitivity analysis to explore the contribution of genetic and environmental factors to the variation in cancer risk among tissues by assuming that tumours may originate from a hierarchy of cells, from stem cells to progenitor cells to differentiated cells [9, 10]. Let *LTCD* denote the true total number of tissue cell divisions per lifetime (from birth to age 74), and *SMN* denote the true somatic mutation number, similarly, the true *LTCD* and *SMN* are almost impossible to be obtained. Let *LTCD*_*0*_ and *SMN*_*0*_ respectively denote the error-prone value of *LSCD* and *SMN* calculated in the laboratory environment. Based on this assumption, we constructed the diagram of relationships between *EH*, *LTCD*_*0*_ (*SMN*_*0*_), *LTCD* (*SMN*), and *LCR* in Figure S1a (b), Additional File 1. For the *i*^*th*^* EH*_*lat*_ (row) in the ranked *LCR* matrix, the measurement error in *LTCD*_*0*_ (*SMN*_*0*_) attributes to its corresponding *EH*_*lat i*_. We calculated *LTCD*_*0*_ of 7 cancer types based on parameters obtained from the Database of Useful Biological Numbers (http://bionumbers.hms.harvard.edu), the supplementary materials in *Wu *et al. [2, 11, 12], and supplementary materials in Tomasetti and Vogelstein [1, 5], the *SMN*_*0*_ of 16 tissues were obtained from the supplementary materials in *Yizhak *et al. [13], see Supplementary Method 3, Additional File 1 for the details. Based on the global-wide ranked *LCR* matrix, we applied the same rationale of modeling strategy by fitting the model $g(LCR_{i} ) = \gamma_{0i} + \gamma_{1i} LTCD_{{0}} + \varepsilon_{{{2}i}}$ and $g(LCR_{i} ) = \lambda_{0i} + \lambda_{1i} SMN_{{0}} + \varepsilon_{{{3}i}}$ by 423 times, respectively, with link function $g(x): = x^{1/5}$ aiming at normalizing variable *LCR*_*i*_, and calculated $R_{i}^{2}$ from each model. We note that the results from the main analysis and this sensitivity analysis also help to identify the cellular origin of cancer. Theoretically speaking, only when the assumed diagram is correct, the contribution of genetic and environmental factors (or stochastic factors) can be measured by the estimated $1 - R_{{}}^{2}$(or $R_{{}}^{2}$), and will show an increased (or decreased) trend as *EH*_*lat*_ changed from good to bad.

Additionally, our modelling strategy assumes that the *LCR*obtained from cancer registries is not affected by screening activity. However, cancer incidence is expected to increase when cancer screening is introduced [14]. In practice, some common cancers (e.g., colorectal and breast cancer) have their own screening tests, and some types of cancer currently do not have an effective screening method (https://www.cancer.net/navigating-cancer-care/prevention-and-healthy-living/cancer-screening). Besides, the quality and coverage of screening activity also vary in different registries. By taking screening into consideration, the differences in observed *LCR* of different cancer types in each registry and observed *LCR* of each organ-specific cancer in different registries may partly due to screening. Let *LCR*_*T*_ denote the unobserved cancer risk that not affected by screening, and *S* denote the screening tests, we constructed the diagram of relationships between *EH*, *LSCD*_*0*_, *LSCD*, *LCR*_*T*_, *S*, and *LCR* (Figure S2, Additional File 1). Then we performed a sensitivity analysis to examine the impact of screening on the results of our modelling strategy, see Supplementary Method 4, Additional File 1 for the details.

### Lifetime risk of each cancer type explained by genetic and environmental factors

As pointed by some studies, an implicit assumption underpinning the correlation between *LCR* and *LSCD*among tissues is that all cancers are induced by a 1-hit model, i.e., every cell division has an equal chance of giving rise to a cancer, regardless of its history [3]. However, it is acknowledged that carcinogenesis requires a multistep accumulation of DNA alterations [9], moreover, it might be that different cancers are suppressed by different numbers of hits [3]. Therefore, based on the simplest form of the multistage model [3, 15], we provided another modeling strategy to calculate the approximate proportion of lifetime cancer risk of each organ-specific cancer that is due to genetic and environmental factors.

For any tissues we considered in this study, let *ACR*_*j*_ denote the cancer risk with respect to the *j*^*th*^ age interval ($j \in \left\{ {1, \cdots ,15} \right\}$ represent age intervals 0–4, 0–9, …, 0–74, respectively), *EH* be a single variable denoting all the genetic and environmental factors that not exist in the laboratory environment, it may include all lifespan external environmental exposure factors and a few hereditary factors which promote the accumulation of divisions of stem cell in this tissue by acting on stem cells or the stem cell environment, as well as the interaction of these factors (e.g., gene–gene or gene-environment interactions). Let *ASCD*_*j*_ denote the true total number of stem cell divisions in *j*^*th*^ age interval that cannot be obtained, and *ASCD*_*0j*_ denote the error-prone observed value of *ASCD*_*j*_ based on parameters obtained under the laboratory environment. The *ACR*_*j*_ of 17 cancer types were calculated in 423 registries in 68 different countries (global-wide), and 139 counties in Shandong province (Shandong provincial, China), respectively. The *ASCD*_*0j*_ of these tissues were calculated using parameters from the supplementary materials in *Tomasetti and Vogelstein* [1, 5], we note that several tissues (breast, prostate, and skin) have different turnover rates or the total number of stem cells at different ages, see Supplementary Method 5, Additional File 1 for the details.

Taking lung adenocarcinoma as an example to illustrate the rationale of the proposed modeling strategy. Those global-wide *ACR* data of lung adenocarcinoma can be organized into an ${423} \times {15}$ original *ACR* matrix of lung adenocarcinoma, the element $ACR_{ij} ,i \in \left\{ {1, \cdots ,423} \right\},j \in \left\{ {1, \cdots ,15} \right\}$ denotes the *ACR* for the *j*^*th*^ age interval in the *i*^*th*^ registry. For the *j*^*th*^ age interval, *ACR*_*j*_ of lung adenocarcinoma exhibited large differences across the world. According to Fig. 1, the differences were due to the change of *ASCD*_*j*_, which is caused by the change in exposure levels to *EH*. Thus, we first constructed a ranked *ACR* matrix of lung adenocarcinoma by sorting the *ACR* in each column (age interval) in original *ACR* matrix from the lowest to the highest, which can indirectly denote the *EH* of lung adenocarcinoma in each age interval ranked from the best to the worst. Similarly, we denote the level of *EH* (row) in the ranked *ACR* matrix as *EH*_*lat*_, the first row of the ranked matrix was regarded as the optimal *EH*_*lat*_ of lung adenocarcinoma of all age interval in 423 registries, followed by the second optimal *EH*_*lat*_, until to the worst *EH*_*lat*_.

Then the simplest form of multistage model [3, 15] was used to estimate the ranges of basic *ACR*_*j*_,$j \in \left\{ {1, \cdots ,15} \right\}$ of lung adenocarcinoma under the laboratory environment (the details are deferred to the Supplementary Method 6, Additional File 1). For *ACR* in the *j*^*th*^ age interval in the *i*^*th*^* EH*_*lat*_ (row) of the ranked *ACR* matrix of lung adenocarcinoma (*ACR*_*ij*_), we regarded that the risks within the estimated range of basic *ACR*_*j*_ are attributed to random mutations arising during DNA replication in normal stem cells, a few genetic factors that exist in the laboratory environment, as well as random error; while the excess absolute risk beyond that range is attributed to *EH*_*lat i*_. Thus, the contribution of genetic and environmental factors to *ACR*_*ij*_ of lung adenocarcinoma can be approximately measured by the proportions of excess absolute risk.

Let $C_{i}$ denote the contribution of genetic and environmental factors for the *i*^*th*^* EH*_*lat*_ (row) of the ranked *ACR* matrix to the risk of lung adenocarcinoma.

$$C_i=\frac{\sum_{j=1}^{15}\left(\max\{0,ACR_{ij}-A\widehat CR_j^{up}\}\right)^2}{\sum_{j=1}^{15}ACR_{ij}^2}\ ,$$

where $A\hat{C}R_{j}^{up}$ is the estimated upper bound of the range of basic *ACR*_*j*_ in the *j*^*th*^ age interval, i.e., the numerator is the sum of squares of excess absolute risk in each age group in *EH*_*lat i*_, the denominator is the sum of squares of *ACR* in each age group in *EH*_*lat i*_. In addition, we calculated the average contribution of genetic and environmental factors to lung adenocarcinoma:

$$C_{total} = \frac{{\sum\nolimits_{i = 1}^{423} {\sum\nolimits_{j = 1}^{15} {\left( {\max \{ 0,ACR_{ij} - A\hat{C}R_{j}^{up} \} } \right)^{2} } } }}{{\sum\nolimits_{i = 1}^{423} {\sum\nolimits_{j = 1}^{15} {ACR_{ij}^{2} } } }}\ ,$$

i.e., the numerator is the sum of squares of excess absolute risk in each age intervals in each *EH*_*lat*_, the denominator is the sum of squares of *ACR* in each age intervals in each *EH*_*lat*_.

Following this modeling strategy, for each single cancer type, we constructed the ranked *ACR* matrixes using global-wide and Shandong provincial data, respectively, and calculated $C_{i}$ ($i \in \left\{ {1,...,423} \right\}$ for global-wide ranked *ACR* matrix, $i \in \left\{ {1,...,139} \right\}$ for Shandong province ranked *ACR* matrix) and $C_{total}$ in each scope.

All statistical analyses were performed using *R*, version 3.6.0.

## Results

**Fig. 3:**
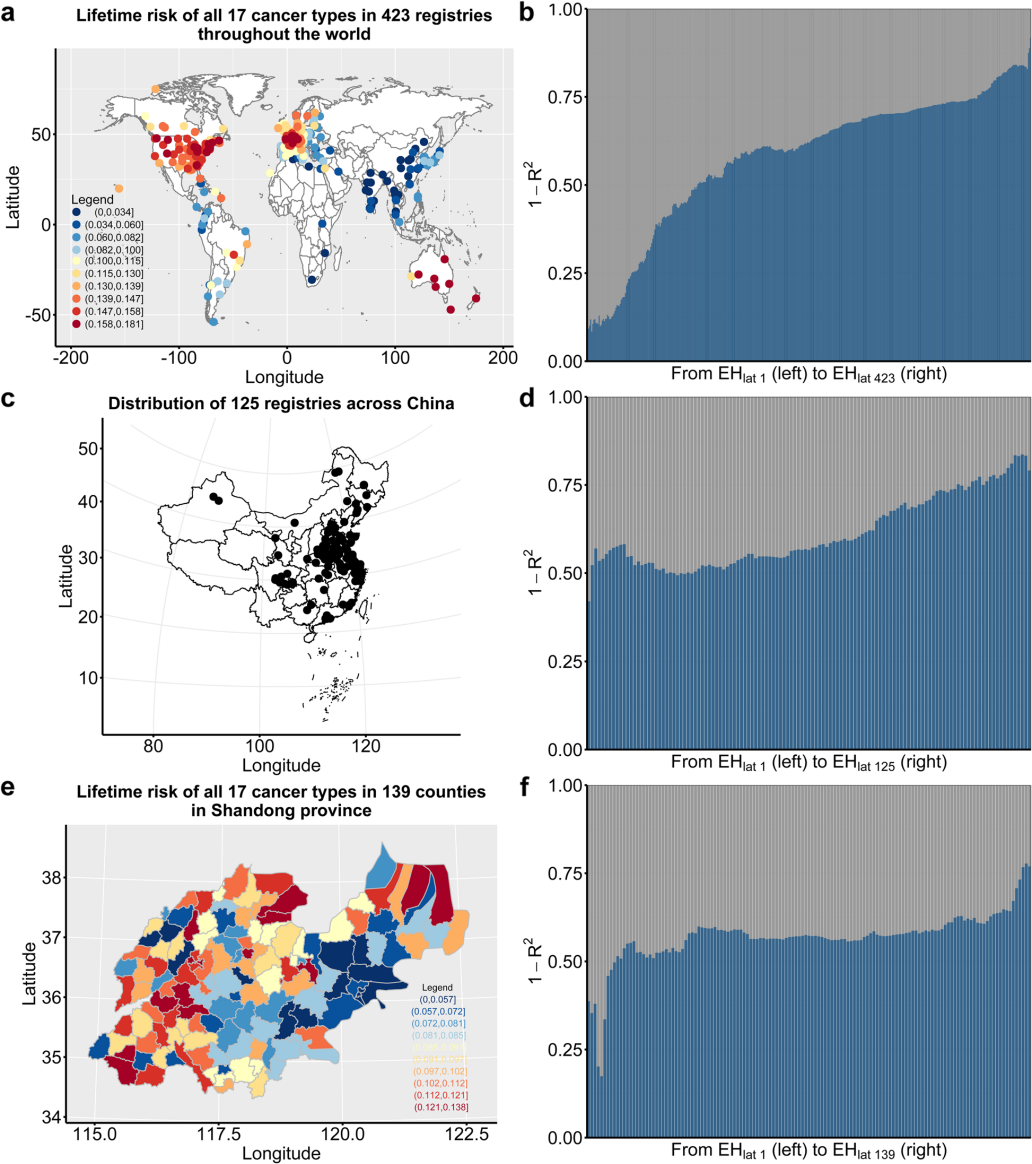
Variation in cancer risk among tissues explained by genetic and environmental factors. **a, c** and **e** The spatial distribution of registries (counties) in global-wide, national-wide in China, and Shandong province, respectively, besides, **a** and **e** show the substantial variation in cancer risks of all 17 cancer types across different registries (counties). [Maps of China and Shandong province were obtained from the Resource and Environment Science and Data Center at http://www.resdc.cn/Default.aspx]. **b, d,** and **f** bar charts of the estimated $1 - R_{i}^{2}$ from curve $g(LCR_{i} ) = \varphi_{i0} + \varphi_{i1} LSCD_{{0}} + \varepsilon_{1i}$ fitting in each *EH*_*lat i*_ in global scope, national scope and provincial scope, respectively, representing the contributions of genetic and environmental factors from the optimal *EH*_*lat*_ (left) to the worst *EH*_*lat*_ (right) to the variation of cancer risks in global scope, national scope, and provincial scope, respectively. *EH*: a single variable denoting all the genetic and environmental factors that do not exist in the laboratory environment; *LSCD*: the true total number of divisions of all stem cells within this tissue per lifetime (from birth to age 74); *LSCD*_*0*_: the error-prone value of *LSCD* calculated using parameters estimated based on results of cell culture from mouse or human tissues in the laboratory environment; *LCR*: the observed lifetime cancer risk. *EH*_*lat i*_: the *i*^*th*^ level (row) of *EH* in the ranked *LCR* matrix

The spatial distribution of the registries (counties) in global-wide, national-wide in China, and Shandong province are shown in Fig. 3a, c, and e, respectively, additionally, Fig. 3a and e also exhibit substantial variation in cancer risk of all 17 cancer types across different registries (counties). The constructed ranked *LCR* matrixes are provided in Table S1-S3, Additional File 2. The bar charts in Fig. 3b, d, and f reveal the contribution of environmental and genetic factors (*EH*) to the differences in cancer risks among different organs (measured by estimated $1 - R_{{}}^{2}$) from the respective optimal *EH*_*lat*_ to the respective worst *EH*_*lat*_ of all 17 cancers in global scope, national scope, and provincial scope. In all three scopes, the contribution of genetic and environmental factors to cancer risks increases as the deteriorates of *EH*_*lat*_. In the respective worst *EH*_*lat*_, the contribution is up to 92% (global scope), 83% (national scope), and 78% (provincial scope), even in the respective optimal *EH*_*lat*_, the contribution of genetic and environmental factors still reaches to 9% (global scope), 42% (national scope), and 35% (provincial scope), see Table S1-S3, Additional File 2 for the details. These results suggest that at least 92% (the maximum $1 - R_{{}}^{2}$ in all three scopes) of the variation in cancer risk among 17 tissues attributes to genetic and environmental factors.

**Fig. 4:**
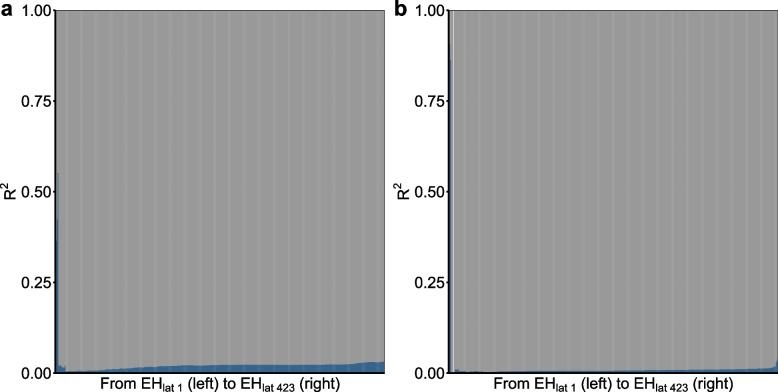
The results of sensitivity analysis assuming that tumours may originate from a hierarchy of cells. **a** bar chart of the estimated $R_{i}^{2}$ from the curve $g(LCR_{i} ) = \gamma_{0i} + \gamma_{1i} LTCD_{{0}} + \varepsilon_{{{2}i}}$ in each *EH*_*lat i*_ ($i = 1, \cdots ,423$) using global-wide ranked *LCR* matrix, representing the contributions of *LTCD*_*0*_ from the optimal *EH*_*lat*_ (left) to the worst *EH*_*lat*_ (right) to the variation of cancer risks; **b** bar chart of the estimated $R_{i}^{2}$ from curve $g(LCR_{i} ) = \lambda_{0i} + \lambda_{1i} SMN_{{0}} + \varepsilon_{{{3}i}}$ in each *EH*_*lat i*_ ($i = 1, \cdots ,423$) using global-wide ranked *LCR* matrix, representing the contributions of *SMN*_*0*_ from the optimal *EH*_*lat*_ (left) to the worst *EH*_*lat*_ (right) to the variation of cancer risks. *LTCD*_*0*_: the error-prone value of the total number of tissue cell divisions per lifetime calculated in the laboratory environment; *SMN*_*0*_: the error-prone value of the somatic mutation number calculated in the laboratory environment; *LCR*: the observed lifetime cancer risk, *EH*_*lat*_: the level of *EH* (row) in the ranked *LCR* matrix

The simulation results in Figure S3, Additional File 1 also indicate that the contribution of environmental and genetic factors to the differences in cancer risks can be approximately and indirectly measured by ${1} - R_{{}}^{2}$, this shows the feasibility of our modelling strategy. Furthermore, the results of sensitivity analysis assuming that tumours originate from all of the stem cell, progenitor cells and differentiated cells using the number of lifetime total cell divisions (*LTCD*_*0*_) or somatic mutation number (*SMN*_*0*_) instead of the total number of stem cell divisions (*LSCD*_*0*_) to calculate the $R_{{}}^{2}$(the proportion of the differences in cancer risk among different tissues can be explained by *LTCD*_*0*_ or *SMN*_*0*_) of each curve are shown in Fig. 4. However, all the estimated $R_{{}}^{2}$ are quite small (median of 0.02 when using *LTCD*_*0*_ and median of 0.007 when using *SMN*_*0*_), and basically do not change as the environment (*EH*_*lat*_) goes from the best to the worst (the detailed data are deferred to the Table S4-S5, Additional File 2). In addition, the results of sensitivity analysis in Figure S4, Additional File 1 show that the screening test will not significantly change the estimated results from the proposed modelling strategy.

**Fig. 5:**
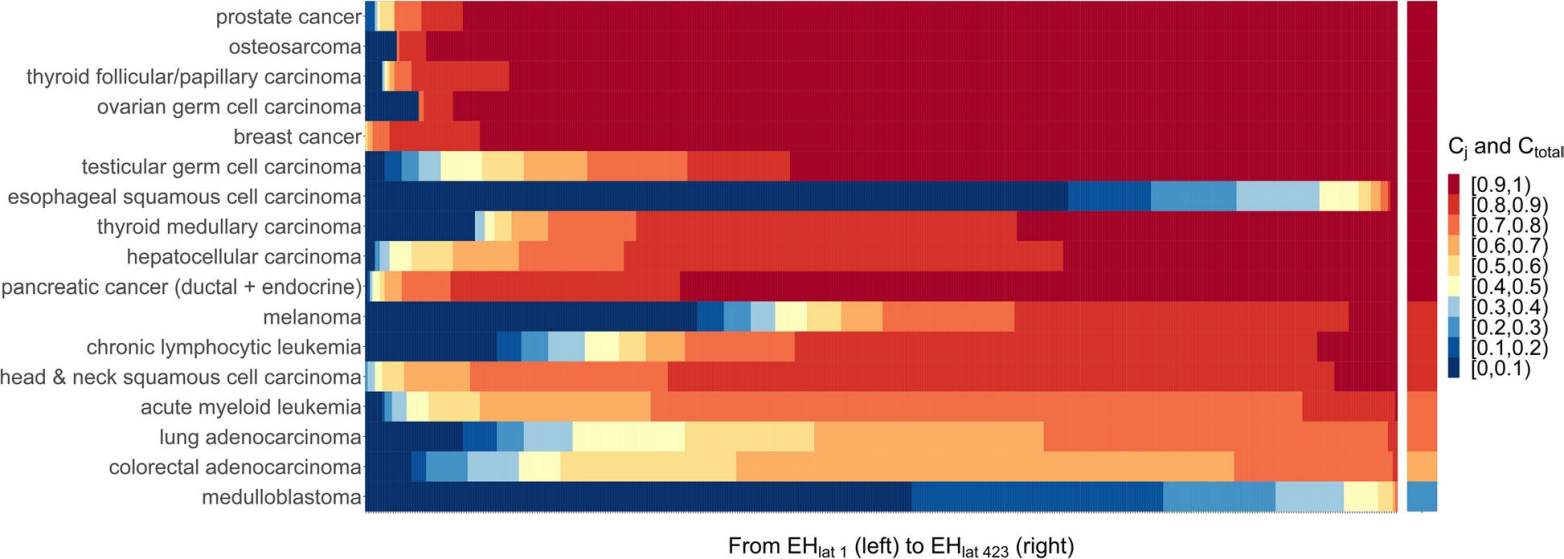
Lifetime risk of each organ-specific cancer explained by genetic and environmental factors in global scope. The left panel denotes the contribution of each *EH*_*lat*_ ($C_{i} ,i = 1, \cdots ,423$) to the risk of each organ-specific cancer, and the lattices from left to right denote $C_{{1}}$(*EH*_*lat 1*_) to $C_{{{423}}}$(*EH*_*lat 423*_), respectively. The right panel denotes the average contribution of genetic and environmental factors ($C_{total}$) to the risk of each organ-specific cancer. *EH*_*lat i*_: the *i*^*th*^ level (row) of *EH* in the ranked *ACR* matrix

**Fig. 6:**
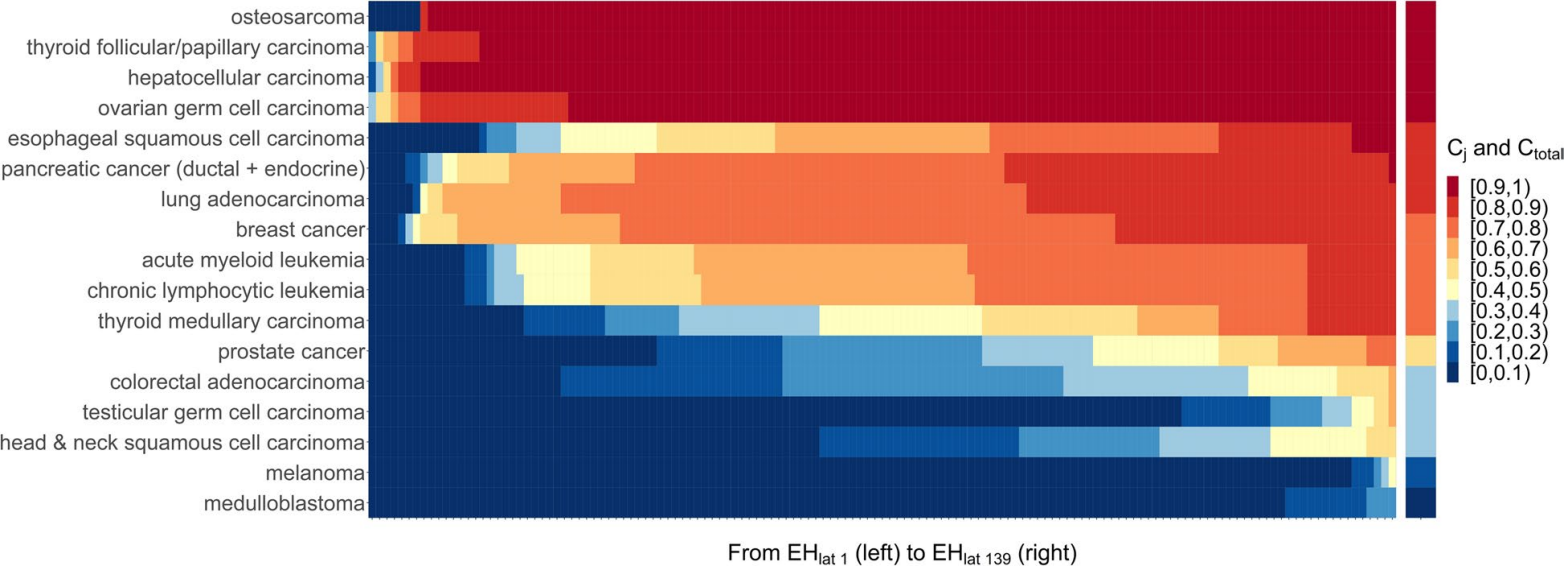
Lifetime risk of each organ-specific cancer explained by genetic and environmental factors in Shandong province. The left panel denotes the contribution of each *EH*_*lat*_ ($C_{i} ,i = 1, \cdots ,{139}$) to the risk of each organ-specific cancer, and the lattices from left to right denote $C_{{1}}$(*EH*_*lat 1*_) to $C_{{{139}}}$ (*EH*_*lat 139*_), respectively. The right panel denotes the average contribution of genetic and environmental factors ($C_{total}$) to the risk of each organ-specific cancer. *EH*_*lat i*_: the *i*^*th*^ level (row) of *EH* in the ranked *ACR* matrix

For each organ-specific cancer, taking lung adenocarcinoma as an example, the constructed ranked *ACR* matrix and the estimated range of basic *ACR* are respectively illustrated by blue dots and yellow dots in Figure S5, Additional File 1. Figures 5 and 6 show the contribution of each *EH*_*lat*_ (row) and the average contribution of genetic and environmental factors to the risk of each organ-specific cancer, using global-wide and Shandong provincial data, respectively. The estimated average contributions of genetic and environmental factors are greater than 80% for 13 of 17 cancer types, and more than 60% for the other 16 cancers except for medulloblastoma (the detailed data of calculated $C_{i}$ and $C_{total}$ were provided in Table S6-S7, Additional File 2). Furthermore, for most cancers, the contribution of genetic and environmental factors increases with the deterioration of environment.

## Discussion

The relative contributions of hereditary and environmental factors versus unavoidable stochastic risk factors are important for understanding the relative merit of primary prevention as opposed to direct efforts to early detection in tissue with higher rates of stem cell divisions. In this study, we provided two distinct modelling strategies through the analysis of measurement error in the estimation of the number of lifetime stem cell divisions to quantitatively evaluate the contribution of genetic and environmental factors to cancer development. Our results show that genetic and environmental factors contribute at least 92% to the variation in cancer risks among 17 tissues. Besides, except for medulloblastoma, the contributions of genetic and environmental factors to the risks of the other 16 cancers are more than 60%. Our results provide additional evidence that genetic and environmental factors play leading roles in cancer risk. Moreover, our sensitivity analysis also provides evidence that mutations occurring in progenitor cells and differentiated cells are less likely to be accumulated and persist enough for cancer to occur, the carcinogenesis is more likely to originates in stem cells.

Our finding that substantial contribution of environmental and genetic carcinogenic factors to cancer development is supported by numerous epidemiology studies. For instance, *Islami *et al*.* [16] estimated that 42.0% of all cancers diagnosed in 2014 among individuals aged 30 years and older in the United States were attributable to major, potentially modifiable risk factors, including cigarette smoking, excess body weight, and alcohol intake, etc. For several cancers, aetiology has been convincingly linked to specific environmental factors resulting in effective cancer prevention (https://www.cancer.gov/about-cancer/causes-prevention/risk), e.g., smoking and lung cancer, ultraviolet radiation and skin cancer, human papillomavirus and cervical cancer, Helicobacter pylori and gastric cancer, and Hepatitis B Virus (HBV) and Hepatitis C Virus and liver cancer [17]. While several specific environmental risk factors for each cancer have been identified, there are still a large number of potential modifiable and thus preventable risk factors are yet to be discovered. These potential risk factors are hard to be specific and clear about to date, but are also included in hereditary and environmental factors we considered in this work.

Our results that mutations occurring in progenitor cells and differentiated cells are less likely to accumulate the DNA alterations required for carcinogenesis is supported by various studies [9, 18, 19]. For instance, *López-Lázaro *et al*.* [18] found a weak positive correlation (mean = 0.14) between the number of gene mutations and cancers risk across 33 tissue types in each of the 5 cancer registries. Besides, several sequencing studies have found zero mutation in genes of many tumor samples [9, 14, 18, 20], this provided evidence that cancer etiology can be better explained by the accumulation of stem cell divisions than by the accumulation of gene mutations. *Shibata *et al*.* [19] found that the small intestines (SI) and colon accumulate similar numbers of replication errors, but SI adenocarcinoma is much rarer than colorectal cancer. This may reflect that SI crypts are smaller and have fewer stem cells than the colon, which reduces the numbers of cells at risk for mutation and perhaps selection efficiency [19].

For organ-specific cancer, we find that the contribution of genetic and environmental factors to the risks of esophageal squamous cell carcinoma and hepatocellular carcinoma across the world is relatively low, while those are generally high across Shandong province. Although the exact carcinogenic factors influencing the former is still unrevealed, the epidemiology evidence suggests that more than 90% of the total number of esophageal cancer cases worldwide occur in China [21]; the latter is caused by the well-known fact of the high acquisition of chronic HBV infection among the local Chinese people [22]. On the opposite, the contribution of genetic and environmental factors to the lifetime risk of melanoma across the world is higher than that across Shandong province. In addition, the contribution of genetic and environmental factors to medulloblastoma is quite low in both the world and Shandong province, which may be caused by the protection function of the blood–brain barrier. To be noted, the contributions of genetic and environmental factors to osteosarcoma, thyroid follicular/papillary carcinoma, ovarian germ cell carcinoma, breast cancer are extremely high in both the world and Shandong province, in fact, it has been reported that substantial burden of hereditary factors or other risk factors plays in these cancers [23, 24]. Besides, glands are assumed to be more susceptible to environmental factors.

## Conclusions

In summary, we found a substantial contribution of genetic and environmental factors on cancer risk through two distinct modelling strategies. Dues to our results, the identification of modifiable extrinsic risk factors for each cancer, including environmental factors and hereditary factors, is highly recommended, and primary prevention in early life-course should be the major focus of cancer prevention.

## Supplementary Information

**Additional file 1: Supplementary Method 1.** Data sources of *LSCD*_*0*_ and *LCR*. **Supplementary Method 2.** Simulation study of the first modelling strategy. **Supplementary Method 3.** Data sources of *LTCD*_*0*_ and *SMN*_*0*_. **Supplementary Method 4.** Sensitivity analysis to examine the impact of screening. **Supplementary Method 5.** The calculation of *ACR* and *ASCD*. **Supplementary Method 6.** The estimation of range of basic *ACR* under the laboratory environment. **Supplementary Figures.** Figure S1-S5.

**Additional file 2: Table S1.** Ranked matrix of *LCR* in global scope and the contribution of the genetic and environmental factors on the variation in cancer risk. **Table S2.** Ranked matrix of *LCR* in national-wide of China and the contribution of the genetic and environmental factors on the variation in cancer risk. **Table S3.** Ranked matrix of *LCR* in counties of Shandong province, China and the contribution of the genetic and environmental factors on the variation in cancer risk. **Table S4.** Ranked matrix of *LCR* in global scope and *R*^*2*^ of *LCR*_*i*_ on *LTCD*_*0*_. **Table S5.** Ranked matrix of *LCR* in global scope and *R*^*2*^ of *LCR*_*i*_ on* SMN*_*0*_*. ***Table S6.** Ranked matrix of *ACR* in global scope and the contribution of genetic and environmental factors for each site-specific cancer. **Table S7.** Ranked matrix of *ACR* in Shandong province and the contribution of genetic and environmental factors for each site-specific cancer.

## Data Availability

The global-wide data of lifetime cancer risk is available in the [the International Agency for Research on Cancer (IARC)] at [http://ci5.iarc.fr/CI5-X/Pages/download.aspx]; The national-wide of China and Shandong provincial, China data of lifetime cancer risk are available from the corresponding author on reasonable request. The data of the number of stem cell divisions can be obtained from [the supplementary materials in Tomasetti and Vogelstein] at [https://doi.org/10.1126/science.1260825 and https://doi.org/10.1126/science.aaf9011]; The data of the number of total cell divisions is available in [the Database of Useful Biological Numbers] at [http://bionumbers.hms.harvard.edu], [supplementary materials in Wu et al.] at [https://doi.org/10.1038/nature16166], and [the supplementary materials in Tomasetti and Vogelstein] at [https://doi.org/10.1126/science.1260825 and https://doi.org/10.1126/science.aaf9011]; the data of somatic mutation number can be obtained from [supplementary materials in Yizhak et al.] at [https://doi.org/10.1126/science.aaw0726].

## Abbreviations

SI: Small Intestines
HBV: Hepatitis B Virus
