# Supplementary material for: Quantifying substantial carcinogenesis of genetic and environmental factors from measurement error in the number of stem cell divisions
Source: BMC Cancer. 2022 Nov 19;22:1194. doi: 10.1186/s12885-022-10219-w (PMC9675110; doi:10.1186/s12885-022-10219-w)
Supplement: Supplementary file 1 — Additional file 1: Supplementary Method 1. Data sources of LSCD0 and LCR. Supplementary Method 2. Simulation study of the first modelling strategy. Supplementary Method 3. Data sources of LTCD0 and SMN0. Supplementary Method 4. Sensitivity analysis to examine the impact of screening. Supplementary Method 5. The calculation of ACR and ASCD. Supplementary Method 6. The estimation of range of basic ACR under the laboratory environment. Supplementary Figures. Figure S1-S5. [file 12885_2022_10219_MOESM1_ESM.pdf]

Additional file 1

Supplementary Method 1: Data sources of $LSCD_0$ and LCR

The error-prone value of the total number of divisions of all stem cells within this tissue per lifetime (from birth to age 74) (*$LSCD_0$*)

Let *s* denote the total number of stem cells found in a fully developed tissue, and *d* denotes the total time of further divisions for each stem cell in the lifetime. [1] The $LSCD_0$ of each tissue equals to the sum of the division number before the tissue has been fully developed ($\sum_{n=1}^{\log_2^s} 2^n$) and the number of further divisions after the tissue has been fully developed during the lifetime (*sd*).[1] *Tomasetti and Vogelstein* [1] has shown that this calculation formula equals to

$$LSCD = s(2+d)-2.$$ [1]

In this study, we consider 17 tissues including that for head and neck squamous cell carcinoma, esophageal squamous cell carcinoma, colorectal adenocarcinoma, hepatocellular carcinoma, pancreatic ductal/endocrine cancer, lung adenocarcinoma, osteosarcoma, melanoma, ovarian germ cell carcinoma, testicular germ cell carcinoma, medulloblastoma, thyroid follicular/papillary carcinoma, thyroid medullary carcinoma, chronic lymphocytic leukemia, acute myeloid leukemia, prostate cancer, and breast cancer. The data of parameter *s* and *d* were obtained from the supplementary materials in *Tomasetti and Vogelstein* [1, 2].

The lifetime cancer risk (*LCR*)

In this study, the *LCR* of 17 cancer types were calculated from global-wide (the same data as *Tomasetti and Vogelstein* [2], covering 423 registers in 68 different countries), national-wide of China (125 registers across China), and Shandong provincial, China (139 counties in Shandong province).

For global-wide *LCR* calculation, the Cancer Incidence in Five Continents Volume X (CI5-X) containing information from 423 registries in 68 different countries about cancers diagnosed from 2003 to 2007 was used. This database is provided by the International Agency for Research on Cancer (IARC) at http://ci5.iarc.fr/CI5-X/Pages/download.aspx. The respective row numbers of 17 organ-specific cancers were 003&075, 024, 042&049, 059, 070, 079, 090, 100, 142, 152, 189, 201&202, 203, 229, 233, 151, 113. This database offered the cancer incidence data with respect to 5-year age intervals. Let d_i denote the number of cases in i^{th} age interval ($i \in \{1,\cdots,15\}$

represent age interval of 0-4, 5-9, 10-14, ..., 70-74), and y_i denote the person-years at risk in i^{th} age interval. Then the *LCR* of each organ-specific cancer was calculated through equation (1) and (2)

$$rate = 5\sum_i \frac{d_i}{y_i}, \quad (1)$$

$$LCR = 1 - \exp(-rate), \quad (2)$$

where *rate* denotes the cumulate rate from ages 0 to 74 of this cancer, see https://publications.iarc.fr/_publications/media/download/3753/609d0d7711047dd76d7f3dbaa25d7f041fcd013e.pdf for more details.

For national-wide of China *LCR* calculation, we used data from 125 registries across China in the year of 2013 obtained from Chinese Cancer Registry Annual Report [3]. This report provides gender-specific cumulate rates (age of 0-74) of totally 25 cancer types. We first obtain the *LCR* of each cancer types using formula (2), then *LCR* for these 17 cancer subtypes were obtained by multiplying the proportion that each cancer subgroup account for (corresponding to 1.8587, 0.861, 0.9854, 0.332, 1, 0.34, 0.2745, 1, 0.0424, 1, 0.03716, 0.95, 0.004, 0.054, 0.199, 1, 1, respectively [3]).

For Shandong provincial *LCR* calculation, 2016 cancer registration database from Shandong Center for Disease Control and Prevention and 2016 population information database from Shandong Provincial Big Data Center were used. The former database provides information about cancer diagnosis in 2016, while the latter contains person-years at risk in each age in each county. These two databases were linked by standard administrative code, then *LCR* of each organ-specific cancer was calculated using formula (1) and (2).

Supplementary Method 2: Simulation study of the first modelling strategy

According to the diagram in **Figure 1**, if we can measure $EH_{lat\ i}$ directly, then the contribution of genetic and environmental factors in i^{th} EH_{lat} can be measured directly by the coefficient of determination from the model $g(LCR_i) = \kappa_{i0} + \kappa_{i1} EH_{lat\ i} + \varepsilon_{4i}$ ($R^2_{EH_{lat\,i}}$). However, $R^2_{EH_{lat\,i}}$ is impossible to be obtained because of the unobservable of $EH_{lat\ i}$. In our modelling strategy, we indicated that the contribution of $EH_{lat\ i}$ to the variation of LCR_i can be measured approximately and indirectly by 1 minus the coefficient of determination from the model $g(LCR_i) = \varphi_{i0} + \varphi_{i1} LSCD_0 + \varepsilon_{1i}$ ($1 - R^2_{LSCD_0}$). We conducted a simulation study to examine this modelling strategy. We considered two scenarios that LCR_i follows a Normal distribution (**Scenario 1**) and non-Normal distribution (**Scenario 2**).

Scenario 1: *LCR_i* follows a normal distribution

For scenario 1, our data (ranked *LCR* matrix) are simulated from the following plausibly realistic data-generating model, corresponding to **Figure 1**. Firstly, we generated $LSCD_0$ from a Uniform distribution

$$LSCD_0 \sim U\left(1\times 10^7, 1\times 10^{12}\right).$$

For i^{th} EH_{lat} (row, $i \in \{1, \cdots, n\}$) in the ranked matrix, we generated $EH_{lat\ i}$ from a Uniform distribution

$$EH_{lat\ i} \sim U\left(100, 100 + \sqrt{12 \cdot \sigma^2_{EH_{lat\ i}}}\right).$$

Then variable $LSCD_i$ was generated based on $LSCD_0$ and $EH_{lat\ i}$:

$$LSCD_i = LSCD_0 + 0.4 EH_{lat\ i} + \varepsilon_0 \ \ with\ \ \varepsilon_0 \sim N\left(0, 100\right).$$

The variance of $EH_{lat\ i}$ ($\sigma^2_{EH_{lat\ i}}$) was chosen to produce a specific $R^2_{True-EH_{lat\,i}}$ value, i.e., the true proportion that the variance of $LSCD_i$ explained by $EH_{lat\ i}$, satisfying the following equation:

$$R^2_{True-EH_{lat\,i}} = \frac{0.16\sigma^2_{EH_{lat\ i}}}{\text{var}\left(LSCD_0\right) + 0.16\sigma^2_{EH_{lat\ i}} + 100} \quad (3)$$

Finally, LCR_i in i^{th} EH_{lat} was generated based on $LSCD_i$:

$$LCR_i = \left(2\times10^{-3}\right) + \left(5\times10^{-14}\right)\cdot LSCD_i + \varepsilon_1 \ with\ \varepsilon_1 \sim U\left(0, \sqrt{12\cdot\sigma_{\varepsilon_1}^2}\right),$$

Similarly, variance $\sigma_{\varepsilon_1}^2$ was chosen to produce a specific $R^2_{True-LSCD_i}$ with

$$R^2_{True-LSCD_i} = \frac{\left(5\times10^{-14}\right)^2 \mathrm{var}(LSCD_i)}{\left(5\times10^{-14}\right)^2 \mathrm{var}(LSCD_i) + \sigma_{\varepsilon_1}^2},$$

we set $R^2_{True-LSCD_i} = 0.95$ for each $i \in \{1,\cdots,n\}$.

We set the number of EH_{lat} level: n=500, and for each EH_{lat}, we considered 17 cancer types. In the generated ranked *LCR* matrix, $LSCD_1$ was generated from equation $LSCD_1 = LSCD_0 + \varepsilon_0 \ with\ \varepsilon_0 \sim N\left(0,100\right)$, while $LSCD_i$ from the second EH_{lat} ($EH_{lat\ 2}$) to the 500th EH_{lat} ($EH_{lat\ 500}$) were generated by varying across $R^2_{True-EH_{lat\,i}}$ in equation (3) from 0.002 to 0.998.

Then, for i^{th} EH_{lat} (row) in the simulated ranked *LCR* matrix, we performed 2000 independent simulations and compared (a) the estimated coefficient of determination from the fitted model $LCR_i = \kappa_{i0} + \kappa_{i1}EH_{lat\,i} + \varepsilon_{4i}$ assuming that $EH_{lat\,i}$ can be measured directly ($\hat{R}^2_{EH_{lat\,i}}$); (b) the 1 minus the coefficient of determination from model $LCR_i = \varphi_{i0} + \varphi_{i1}LSCD_0 + \varepsilon_{1i}$, i.e., our modelling strategy ($1-\hat{R}^2_{LSCD_0}$).

Scenario 2: LCR_i follows a non-normal distribution

For scenario 2, our data are simulated from the following plausibly realistic data-generating model, corresponding to **Figure 1**. First, we generated $LSCD_0$, $EH_{lat\,i}$, and $LSCD_i$ according to the same model in Scenario 1, then we generated LCR_i following

$$\sqrt[5]{LCR_i} = \left(2\times10^{-3}\right) + \left(5\times10^{-14}\right)\cdot LSCD_i + \varepsilon_1 \ with\ \varepsilon_1 \sim U\left(0, 0.006\right)$$

We set the number of EH_{lat} level: n=500, and for each EH_{lat}, we considered 17 cancer types. In the generated ranked *LCR* matrix, $LSCD_1$ was generated from equation $LSCD_1 = LSCD_0 + \varepsilon_0 \ with\ \varepsilon_0 \sim N\left(0,100\right)$, while $LSCD_i$ from the second EH_{lat} ($EH_{lat\ 2}$) to the

500th EH_{lat} ($EH_{lat\ 500}$) were generated by varying across $R^2_{True-EH_{lat\,i}}$ in equation (3) from 0.002 to 0.998.

Then, for i^{th} EH_{lat} (row) in the simulated ranked *LCR* matrix, we performed 2000 independent simulations and compared (a) the estimated coefficient of determination from the fitted model $g(LCR_i) = \kappa_{i0} + \kappa_{i1} EH_{lat\ i} + \varepsilon_{4i}$ with $g(x) = \sqrt[5]{x}$ (chosen to normalize LCR_i) assuming that $EH_{lat\ i}$ can be measured derictly ($\hat{R}^2_{EH_{lat\,i}}$); (b) the 1 minus the estimated coefficient of determination from model $g(LCR_i) = \varphi_{i0} + \varphi_{i1} LSCD_0 + \varepsilon_{1i}$, i.e., our modelling strategy ($1-\hat{R}^2_{LSCD_0}$).

Supplementary Method 3: Data sources of $LTCD_0$ **and** *SMN_0*

The error-prone total number of tissue cell divisions per lifetime (from birth to age 74) $LTCD_0$ of seven cancers, including colorectal adenocarcinoma, hepatocellular carcinoma, lung adenocarcinoma, osteosarcoma, testicular germ cell carcinoma, prostate cancer, breast cancer (the details about the cellular turnover rates for other ten tissues are not available) were calculated through

$$LTCD = \left[(74 \times 365) / v \right] \times s^*,$$

where v denotes the tissue turnover rate per cell per day, obtained from the Database of Useful Biological Numbers (http://bionumbers.hms.harvard.edu) as well as supplementary materials in *Wu et al.* [4-6]; and s^* represents total tissue cell numbers, acquired from supplementary materials in *Tomasetti and Vogelstein* [1,2].

The error-prone somatic mutation number SMN_0 of 16 tissues (excluding tissue of osteosarcoma) were obtained from the supplementary materials in *Yizhak et al.* [7]. This study applied RNA-MuTect to 6707 samples against their matched-blood DNA, which spanned 29 human tissues and 488 individuals, and detected 8870 somatic mutations in 37% of the samples. [7] The maximal somatic mutation number of each tissue was used as SMN_0 in our study.

Supplementary Method 4: Sensitivity analysis to examine the impact of screening

We performed a simulation study to examine the impact of screening on the results of our modelling strategy. Our data are simulated from the following plausibly realistic data-generating model, corresponding to **Figure S2**. First, $LSCD_0$ of 17 tissues were calculated based on parameters from the supplementary materials in *Tomasetti and Vogelstein* [1,2], then we generated $EH_{lat\,i}$ and $LSCD_i$ according to the same model in Scenario 1 in **Supplementary Method 2**, then we generated LCR_{Ti}, S, and LCR in turn as follows:

$$\sqrt[5]{LCR_{Ti}} = \left(2\times10^{-3}\right)+\left(5\times10^{-14}\right)\cdot LSCD_i+\varepsilon_1 \; with \; \varepsilon_1 \sim U\left(0,0.006\right),$$
$$S=\chi\cdot LCR_{Ti}+\left(1\times10^{-17}\right)\cdot EH_{lat\;i}+\varepsilon_s \; with \; \varepsilon_s \sim U\left(1\times10^{-6},5\times10^{-6}\right),$$
$$LCR_i = LCR_{Ti}+0.85\cdot S+\varepsilon_{lcr} \; with \; \varepsilon_{lcr}\sim U\left(1\times10^{-3},2\times10^{-3}\right).$$

We set the number of EH_{lat} level: n=500, and for each EH_{lat}, we considered 17 cancer types. To be noted, $LSCD_1$ was generated from equation $LSCD_1 = LSCD_0+\varepsilon_0 \; with \; \varepsilon_0 \sim N\left(0,100\right)$, while $LSCD_i$ from the second EH_{lat} ($EH_{lat\,2}$) to the 500th EH_{lat} ($EH_{lat\,500}$) were generated by varying across $R^2_{True-EH_{lat\,i}}$ in equation (3) from 0.002 to 0.998.

We focus on two cases with the effect of LCR_{Ti} on S (parameter χ) equals to 0.05 (**case 1**) and 0.3 (**case 2**), respectively, with other parameters fixed in their initial values. Then for each case, for i^{th} EH_{lat} (row) in the simulated ranked LCR matrix, we performed 2000 independent simulations and compared (a) the estimated coefficient of determination from the fitted model $g(LCR_{Ti})=\kappa_{i0}+\kappa_{i1}EH_{lat\;i}+\varepsilon_{4i}$ with $g(x)=\sqrt[5]{x}$ (chosen to normalize LCR_i) assuming that $EH_{lat\,i}$ and LCR_{Ti} can be measured directly ($\hat{R}^2_{EH_{lat\,i}-True}$), i.e., the true contribution of $EH_{lat\,i}$ to the variation in cancer risk among tissues when screening is absent; (b) the 1 minus the estimated coefficient of determination from model $g(LCR_{Ti})=\varphi_{i0}+\varphi_{i1}LSCD_0+\varepsilon_{1i}$ assuming that LCR_{Ti} can be measured directly ($1-\hat{R}^2_{LSCD_0-True}$), i.e., the contribution calculated using the proposed strategy when screening is absent; (c) the estimated coefficient of determination from the fitted model $g(LCR_i)=\kappa_{i0}+\kappa_{i1}EH_{lat\;i}+\varepsilon_{4i}$ with $g(x)=\sqrt[5]{x}$ (chosen to normalize LCR_i) assuming that $EH_{lat\,i}$ can be measured directly ($\hat{R}^2_{EH_{lat\,i}}$), i.e., the contribution of $EH_{lat\,i}$ when screening is present; (d)

the 1 minus the estimated coefficient of determination from model $g(LCR_i) = \varphi_{i0} + \varphi_{i1} LSCD_0 + \varepsilon_{1i}$ ($1 - \hat{R}^2_{LSCD_0}$), i.e., the contribution calculated using the proposed strategy when screening is present.

Supplementary Method 5: The calculation of ACR and ASCD

The cancer risk with respect to the jth age interval (ACR_j, $j \in \{1, \cdots, 15\}$, represent age intervals 0-4, 0-9, …, 0-74, respectively) were calculated from global-wide (423 registers in 68 different countries) and Shandong provincial, China (139 counties in Shandong province). The observed total number of stem cell divisions in j^{th} age interval $ASCD_{0j}$ were calculated through

$$ASCD_{0j} = s(2 + d_j) - 2,$$ [1]

where d_j denotes the total time of further divisions for each stem cell in j^{th} age interval, parameters s and d_j were obtained from supplementary materials in *Tomasetti and Vogelstein* [1-2]. During the calculation process of $ASCD_{0j}$, several tissues (breast, prostate, and skin) have different turnover rate or total number of stem cell at different age:

Breast tissue. The turnover rate of human breast varies between 22 days (in 20-year old individuals) and 147 days (in 40-year old individuals). [6] Therefore, we assumed the average turnover rate was 22 days when calculated $ASCD_{0j}$ of breast cancer in age group 0-4, 0-9, …, 0-29 years. The $ASCD_{0j}$ in age group of 0-34, 0-39, …, 0-74 were the sum of $ASCD_{0j}$ in age group 0-29 years, and the addition division number after age 29 with turnover rate of 147 days.

Prostate tissue. The human prostate has a weight that increases with age: 28.2 g in 40-49 year old individuals, 30.8 g in 50-59 year old individuals, 35 g in 60-70 year old individuals [8], and 46.2 g in those older than 75 years [9]. Thus, we assume parameter s equals to 1.974×10^8 for age group of 0-4, 0-9, …, 0-49. And $ASCD_{0j}$ of prostate cancer in age group of 0-54, 0-59 was the sum of $ASCD_{0j}$ in age group 0-49 years, and the stem cell division number after age 49 with parameter s equals to 2.156×10^8; $ASCD_{0j}$ in age group of 0-64, 0-69, and 0-74 was the sum of $ASCD_{0j}$ in 0-59, and the addition division number after age 59 with parameter s equals to 2.45×10^8.

Skin. the human body surface-area of the skin increases with age. Thus, we assume s is 1.08×10^9 for age group of 0-4 and 0-9. $ASCD_{0j}$ of melanoma in age group of 0-14 and 0-19 were the sum of $ASCD_{0j}$ in 0-9 and the division times after age 9 with parameter s equals to 1.86×10^9; $ASCD_{0j}$ in other age groups were the sum of $ASCD_{0j}$ in age interval of 0-19, and the division times after age 19 with parameter s equals to 2.01×10^9.

Supplementary Method 6: The estimation of range of basic ACR under the laboratory environment

The simplest form of multistage model can be represented by

$$ACR_j = s(d_j \cdot \mu)^M \quad (4),$$

where ACR_j denotes the cumulate riskin j^{th} age interval, *s* denotes the total number of stem cells found in a fully developed tissue, and d_j denotes the total time of further divisions for each stem cell in j^{th} age interval, μ denotes the somatic mutation rate, which is usually assumed to be constant across tissues, and *M* is the number of hits required to initiate one cancer .[10,11] In this function, μ and *M* are two unknown parameters to be estimated.

Since the obtained *s* and d_j of each organ-specific cancer were estimated based on the laboratory environment, ACR_j in equation (4) should be cancer risk in laboratory environment (ACR_{0j}). We assume that ACR_{0j} for each cancer type approximately equals to the minimum non-zero value of ACR_j of that cancer in global-wide and Shandong provincial ranked *ACR* matrixes. Therefore, accompany by estimated s and d_j, the ACR_{0j} of 17 organ-specific cancers were first used to estimate parameters μ and M . Those values of μ which makes the estimated $\hat{M}$ (rounded to the nearest integer) of all 17 cancer types keeping at the reasonable range of $3 \le \hat{M} \le 7$ were considered as the range of μ . [10-12] Through the calculation, the estimated range of $\hat{\mu}$ was narrow: $\left[2 \times 10^{-6}, 5.7 \times 10^{-6}\right]$, which is broadly consistent to that calculated by *Nunney et al.* [10].

Finally, in virtue of the uncertainty of parameter μ and M , we estimated the ranges of basic ACR_j, $j \in \{1, \cdots, 15\}$ of each organ specific cancer under the laboratory environment. Taking lung adenocarcinoma as an example, the estimated ranges of basic ACR_j for each *j* was obtained by substitute *s*, d_j, each $\hat{\mu}$ in the estimated range and its corresponding $\hat{M}$ into formular (4) (yellow dots in **Figure S5, Additional File 1**).

Supplementary Figures

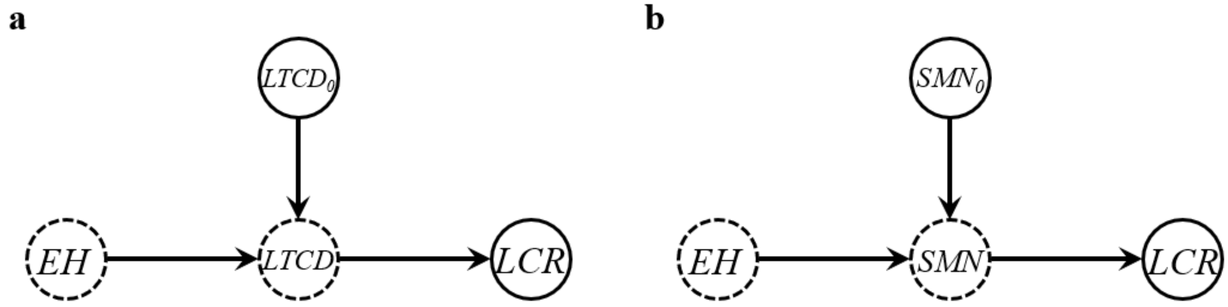

Figure S1. Diagram of relationships between *EH*, *$LTCD_0$* (*SMN_0*), *LTCD* (*SMN*), and *LCR*. the dotted node denotes the unobserved variable, and the solid node represents the observed variable. *EH*: a single variable denoting all the genetic and environmental factors that do not exist in the laboratory environment; *LTCD*: the true total number of tissue cell divisions per lifetime (from birth to age 74); *$LTCD_0$*: the error-prone value of *LSCD* calculated in laboratory environment; *SMN*: the true somatic mutation number; *SMN_0*: the error-prone value of *SMN* calculated in laboratory environment. *LCR*: the lifetime cancer risk.

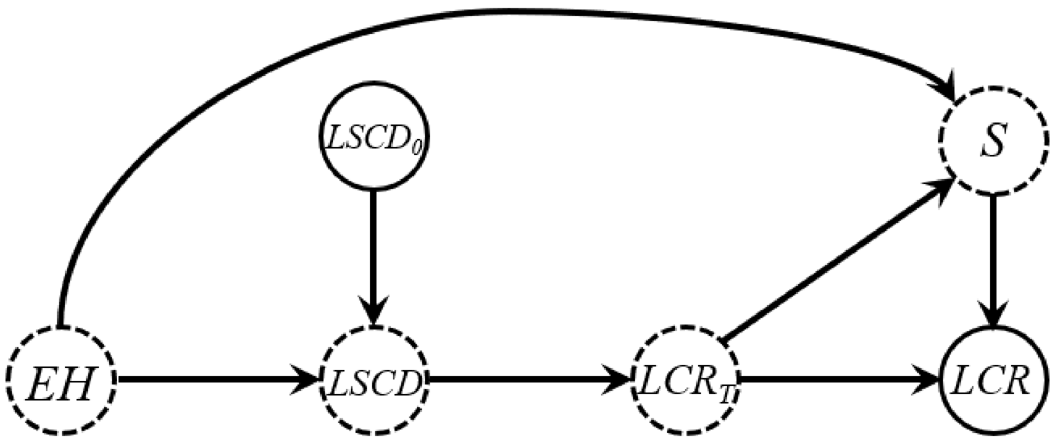

Figure S2. Diagram of relationships between *EH*, *$LSCD_0$*, *LSCD*, *LCR_T*, *S*, and *LCR*. The dotted node denotes the unobserved variable, and the solid node represents the observed variable. *EH*: a single variable denoting all the genetic and environmental factors that do not exist in the laboratory environment; *LSCD*: the true total number of divisions of all stem cells within this tissue per lifetime (from birth to age 74); *$LTCD_0$*: the error-prone value of *LSCD* calculated using parameters estimated based on results of cell culture from mouse or human tissues in the laboratory environment; *LCR_T*: the unobserved cancer risk that not affected by screening; *S*: the screening tests. *LCR*: the observed lifetime cancer risk.

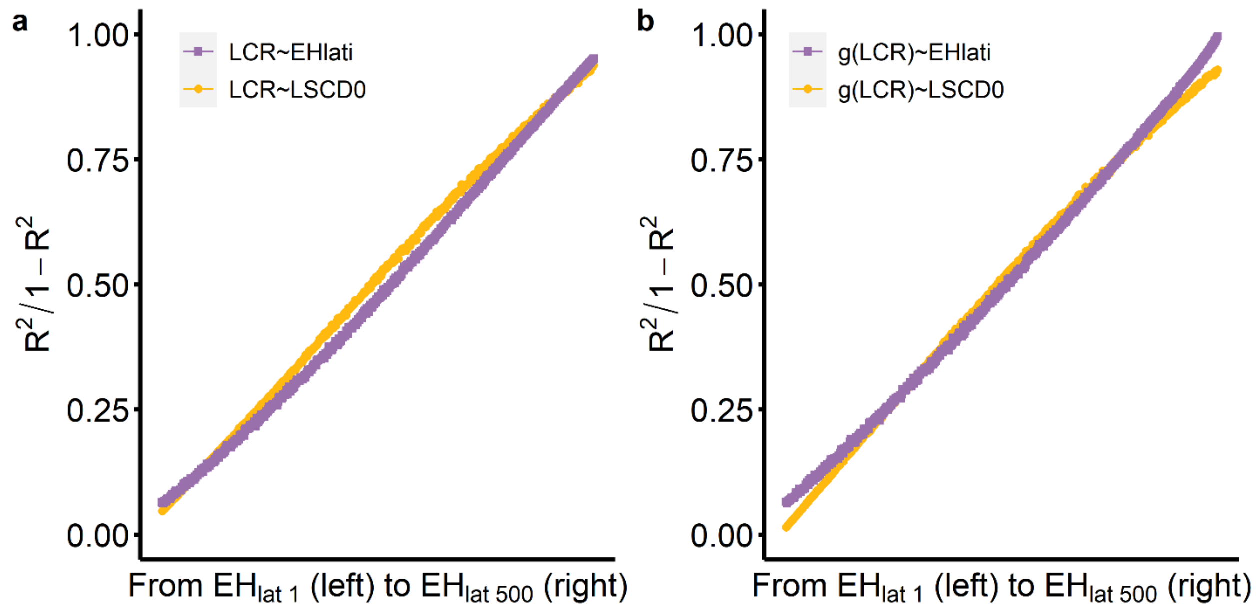

Figure S3. The results of simulation study of the first modelling strategy. a: Simulation results of scenario 1 (LCR follows a normal distribution). **b**: Simulation results of scenario 2 (LCR follows a non-normal distribution). The purple line denotes the estimated coefficient of determination from the fitted model $LCR_i = \kappa_{i0} + \kappa_{i1} EH_{lat\,i} + \varepsilon_{4i}$ assuming that $EH_{lat\,i}$ can be measured directly ($\hat{R}^2_{EH_{lat\,i}}$), and the yellow line denotes the 1 minus the coefficient of determination from model $LCR_i = \varphi_{i0} + \varphi_{i1} LSCD_0 + \varepsilon_{1i}$, i.e., our modelling strategy ($1 - \hat{R}^2_{LSCD_0}$).

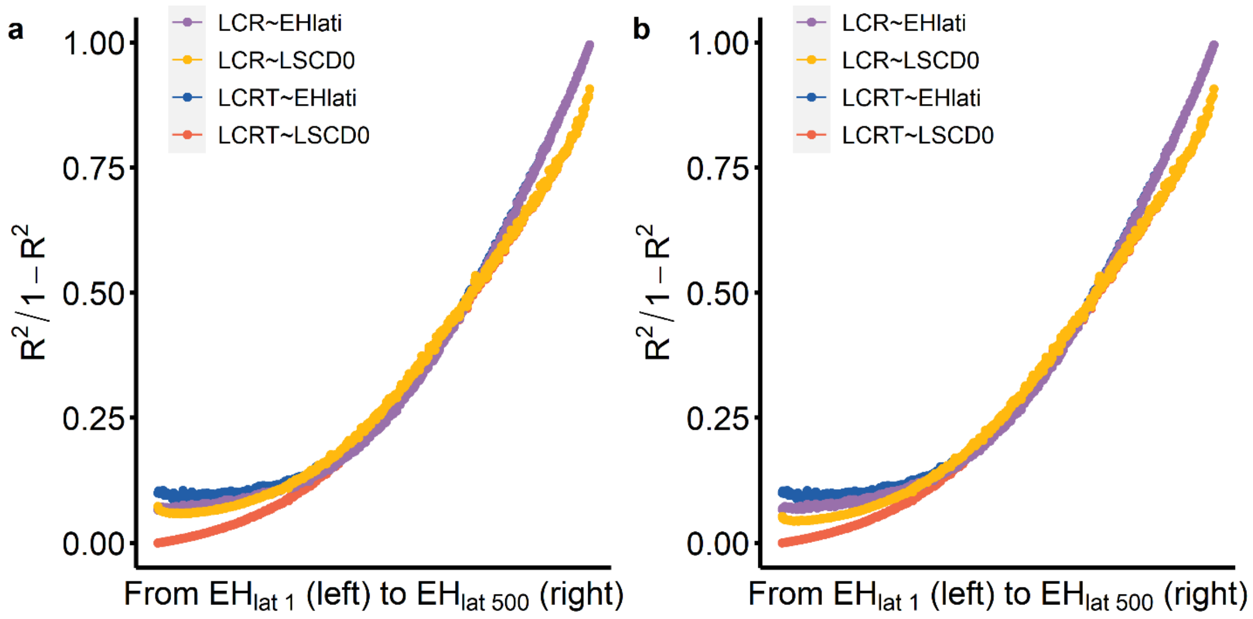

Figure S4. The results of sensitivity analysis to examine the impact of screening on the results of our modelling strategy. **a**: Simulation results of case 1 (the effect of LCR_{Ti} on S equals to 0.05). **b**: Simulation results of case 2 (the effect of LCR_{Ti} on S equals to 0.3). The purple line denotes the estimated coefficient of determination from the fitted model $g(LCR_i) = \kappa_{i0} + \kappa_{i1}EH_{lat\,i} + \varepsilon_{4i}$ with $g(x) = \sqrt[5]{x}$ (chosen to normalize LCR_i) assuming that $EH_{lat\,i}$ can be measured directly ($\hat{R}^2_{EH_{lat\,i}}$),the yellow line denotes the 1 minus the estimated coefficient of determination from model $g(LCR_i) = \varphi_{i0} + \varphi_{i1}LSCD_0 + \varepsilon_{1i}$ ($1-\hat{R}^2_{LSCD_0}$), the blue line denotes the estimated coefficient of determination from the fitted model $g(LCR_{Ti}) = \kappa_{i0} + \kappa_{i1}EH_{lat\,i} + \varepsilon_{4i}$ with $g(x) = \sqrt[5]{x}$ (chosen to normalize LCR_i) assuming that $EH_{lat\,i}$ and LCR_{Ti} can be measured directly ($\hat{R}^2_{EH_{lat\,i}-True}$), and the red line denotes the 1 minus the estimated coefficient of determination from model $g(LCR_{Ti}) = \varphi_{i0} + \varphi_{i1}LSCD_0 + \varepsilon_{1i}$ assuming that LCR_{Ti} can be measured directly ($1-\hat{R}^2_{LSCD_0-True}$).

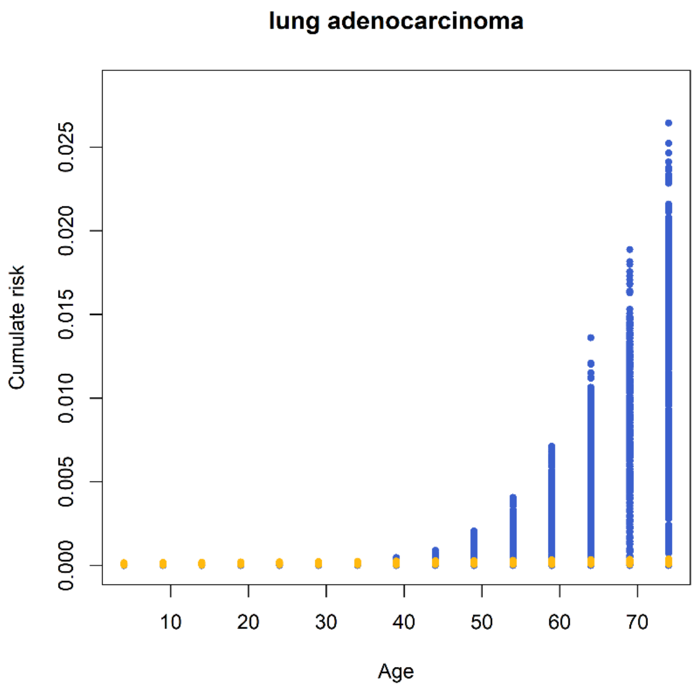

Figure S5. Scatter diagram of the ranked *ACR* matrix and the estimated range of basic *ACR* for lung adenocarcinoma. The ranked *ACR* matrix (including age interval of 0-4, 0-9, …, 0-74) was illustrated by blue dots, and the estimated range of basic *ACR* was illustrated by yellow dots. *ACR*: the cancer risk with respect to the specific age interval.

References

1. Tomasetti C, Vogelstein B. Variation in cancer risk among tissues can be explained by the number of stem cell divisions. Science. 2015;347:78-81.

2. Tomasetti C, Li L, Vogelstein B. Stem cell divisions, somatic mutations, cancer etiology, and cancer prevention. Science. 2017;355:1330-1334.

3. He J, Ping Z, Chen W. 2016 Chinese cancer registry annual report. Tsinghua university press, Beijing, China; 2017.

4. Wu S, Powers S, Zhu W, Hannun YA. Substantial contribution of extrinsic risk factors to cancer development. Nature. 2016;529:43-47.

5. Berges RR, Vukanovic J, Epstein JI, CarMichel M, Cisek L, Johnson DE, et al. Implication of cell kinetic changes during the progression of human prostatic cancer. Clin Cancer Res. 1995;1:473-480.

6. Meyer JS. Cell proliferation in normal human breast ducts, fibroadenomas, and other ductal hyperplasias measured by nuclear labeling with tritiated thymidine. Effects of menstrual phase, age, and oral contraceptive hormones. Hum Pathol. 1977;8:67-81.

7. Yizhak K, Aguet F, Kim J, Hess JM, Kübler K, Grimsby J, et al. RNA sequence analysis reveals macroscopic somatic clonal expansion across normal tissues. Science. 2019;364:eaaw0726.

8. Zhang SJ, Qian HN, Zhao Y, Sun K, Wang HQ, Liang GQ, et al. Relationship between age and prostate size. Asian J Androl. 2013;15:116–120.

9. Bosch JL, Tilling K, Bohnen AM, Bangma CH, Donovan JL. Establishing normal reference ranges for prostate volume change with age in the population based Krimpen-study: Prediction of future prostate volume in individual men. Prostate. 2007;67:1816–1824.

10. Nunney L, Muir B. Peto's paradox and the hallmarks of cancer: constructing an evolutionary framework for understanding the incidence of cancer. Philos Trans R Soc Lond B Biol Sci. 2015;370:20150161.

11. Nunney L. Lineage selection and the evolution of multistage carcinogenesis. Proc Biol Sci. 1999;266:493-498.

12. Armitage P, Doll R. The age distribution of cancer and a multi-stage theory of carcinogenesis. Br J Cancer. 2004;91:1983-1989.

Additional file 2

Table.S1: Ranked matrix of *LCR* in global scope and the contribution of the genetic and environmental factors on the variation in cancer risk

Table.S2: Ranked matrix of *LCR* in national-wide of China and the contribution of the genetic and environmental factors on the variation in cancer risk

Table.S3: Ranked matrix of *LCR* in counties of Shandong province, China and the contribution of the genetic and environmental factors on the variation in cancer risk

Table.S4: Ranked matrix of *LCR* in global scope and R^2 of LCR_i on $LTCD_0$

Table.S5: Ranked matrix of *LCR* in global scope and R^2 of LCR_i on SMN_0

Table.S6: Ranked matrix of *ACR* in global scope and the contribution of genetic and environmental factors for each site-specific cancer

Table.S7: Ranked matrix of *ACR* in Shandong province and the contribution of genetic and environmental factors for each site-specific cancer
